# Lower gastrointestinal bleeding—Computed Tomographic Angiography, Colonoscopy or both?

**DOI:** 10.1186/s13017-016-0112-3

**Published:** 2017-01-03

**Authors:** Daniel Clerc, Fabian Grass, Markus Schäfer, Alban Denys, Nicolas Demartines, Martin Hübner

**Affiliations:** 1 Department of Visceral Surgery, University Hospital of Lausanne (CHUV), Lausanne, Switzerland; 2 Department of Interventional Radiology, University Hospital of Lausanne (CHUV), Lausanne, Switzerland

**Keywords:** Gastrointestinal hemorrhage, Colonoscopy, Computed tomographic angiography, Endoscopy

## Abstract

**Background:**

Lower endoscopy (LE) is the standard diagnostic modality for lower gastrointestinal bleeding (LGIB). Conversely, computed tomographic angiography (CTA) offers an immediate non-invasive diagnosis visualizing the entire gastrointestinal tract. The aim of this study was to compare these 2 modalities with regards to diagnostic value and bleeding control.

**Methods:**

Tertiary center retrospective analysis of consecutive patients admitted for LGIB between 2006 and 2012. Comparison of patients with LE *vs.* CTA as first exam, respectively, with emphasis on diagnostic accuracy and bleeding control.

**Results:**

Final analysis included 183 patients; 122 (66.7%) had LE first, while 32 (17.5%) had CTA; 29 (15.8%) had neither of both exams. Median time to CTA was shorter compared to LE (3 (IQR = 8.2) *vs.* 22 (IQR = 36.9) hours, *P* < 0.001). Active bleeding was identified in 31% with CTA *vs.* 15% with LE (*P* = 0.031); a non-actively bleeding source was found by CTA and LE in 22 *vs.* 31%, respectively (*P* = 0.305). Bleeding control required endoscopy in 19%, surgery in 14% and embolization in 1.6%, while 66% were treated conservatively. Post-interventional bleeding was mostly controlled by endoscopic therapy (57%). 80% of patients with active bleeding on CTA required surgery.

**Conclusions:**

Post-interventional LGIB was effectively addressed by LE. For other causes of LGIB, CTA was efficient, and more available than colonoscopy. Treatment was conservative for most patients. In case of active bleeding, CTA could localize the bleeding source and predict the need for surgery.

## Background

Lower gastrointestinal bleeding (LGIB) is a common clinical problem, representing 20 to 30% of patients presenting with gastrointestinal bleeding [1, 2]. LGIB incidence is increasing over time, as it is associated with older age and pre-existing comorbidities [3]. Very distal bleeding, e.g. due to hemorrhoids and low rectal cancer, is rather easy to diagnose, but bleeding from the colon and small bowel remains a diagnostic challenge. According to recent guidelines, hemodynamic stabilization and resuscitation must be performed prior to search any bleeding source. While nasogastric lavage and/or esophagogastroduodenoscopy can be considered to rule out upper gastrointestinal bleeding for patients presenting severe hematochezia, lower endoscopy (LE) is the preferred diagnostic approach for LGIB [1, 4]. Nevertheless, computed tomographic angiography (CTA) for evaluation of GI bleeding is increasingly used and may challenges lower endoscopy as most appropriate tool. This non-invasive diagnostic modality is readily available in most hospitals and can be rapidly performed without any bowel preparation. Reported sensitivity and specificity rates are 86 and 95%, respectively and bleeding as low as 0.4ml/min can be detected [5, 6].

So far, there is only limited evidence on the routine use of CTA in the initial management of LGIB. Its accuracy compared to LE remains unclear; and subsequently, its use as a first-line diagnostic modality is not considered in current management algorithms [1, 4].

This current study aimed to compare the accuracy of CTA and colonoscopy in the diagnosis of LGIB and their influence on bleeding control.

## Methods

Retrospective cohort study conducted at a tertiary care academic center. The study was approved by the local ethics committee (protocol 389/12) performed according the STROBE recommendations, and registered under www.researchregistry.com (# 726).

### Patients and data collection

All consecutive patients admitted for LGIB to the department of visceral surgery between January 2006 and December 2012 were potentially eligible. The following patients were excluded from analysis: (I) proctological bleeding, (II) LGIB not being the primary cause of admission, and (III) patients with LGIB admitted for elective surgery. Patients underwent primary evaluation at the emergency department by emergency physicians and by a gastrointestinal consultant surgeon before admission to the visceral surgery department. The initial diagnostic modality (LE or CTA) was defined by the physician in charge of the patient.

LE was defined as a flexible lower endoscopy performed by a gastroenterologist. Procedures were performed by an attending, junior staff or resident gastroenterologist according to the policy of a Swiss university hospital. Resident procedures were supervised by a board-certified gastroenterologist. Gastroenterology consultant is available 24/7 in our institution, with in-hospital presence during daytime, and available within 30 min during the nightshift. Type of bowel preparation, if any, was upon the choice of the gastroenterologist performing the procedure. A standard flexible colonoscope was used for all procedures. Conscious sedation or general anesthesia was used depending upon patient’s general condition. Whenever possible, examination was performed up to the ileo-caecal valve. Exam findings were classified as positive with actively bleeding lesion, positive with non-actively bleeding lesion or inconclusive. LE was defined inconclusive when no lesion was detected regardless of the quality of preparation and the amount of blood clots present in the endoscopic view.

For actively bleeding lesions or non-actively bleeding lesions with high risk of re-bleeding, endoscopic therapy was directly applied with clips, adrenaline infusion or thermal probe depending on the gastroenterologist’s preference.

CTA was defined as a contrast-enhanced abdominal and pelvic CT scan performed in a triphasic acquisition. First, a native acquisition was followed by contrast-enhanced arterial phase after a bolus injection of 100ml contrast media (300mg iodine/ml, 4ml/s) with automatic triggering (collimation 16 × 0.625mm; pitch 1.75; table speed 35mm/s). The venous acquisition was performed 70–80 s later. Exam findings were classified as positive with active bleeding when CTA showed intraluminal contrast material extravasation. CTA were defined positive without active bleeding when the cause of bleeding was spotted without contrast material extravasation. CTA were defined inconclusive when no cause of bleeding was found.

The time spent from the admission to the emergency room to the execution of the diagnostic exams was recorded. First hemodynamic parameters (heart rate (HR), blood pressure) and hemoglobin (Hb) values recorded upon admission were retrieved. The shock index (SI), defined as the ratio of heart rate to systolic blood pressure, and the mean arterial pressure (MAP), were calculated.

The cause of bleeding was classified in six categories: small bowel, diverticular, colorectal neoplasia, colorectal lesion, post-interventional, and unknown location. Small bowel bleeding was defined as the source of bleeding arising from the ligament of Treitz to the ileo-caecal valve. Diverticular bleeding was defined as the source of bleeding being related to a colonic diverticular disease. A colorectal neoplasia was defined as benign or malignant lesion being the cause of bleeding. Non-neoplastic, non-diverticular colorectal lesions being the source of bleeding (colitis, colonic ulcers or angiodysplasias) were grouped in the colorectal lesion category. Post-interventional bleeding was defined as a bleeding occurring following endoscopic or surgical procedure. Bleeding of unknown location was defined when the cause of bleeding was not found during hospital stay. The control of bleeding was recorded according to the last therapeutic intervention performed (surgery, angio-embolization, endoscopic intervention or conservative).

Patient’s characteristics, information on the performed diagnostic procedures and clinical outcomes were defined *a priori*. All eligible patients were collected by ICD codes. Data was retrieved by retrospective chart review and entered in a coded computerized database. Patients were stratified according to the first diagnostic exam performed at hospital admission, i.e. LE, CTA or none of both exams.

### Statistical analysis

Descriptive statistics for categorical variables were reported as frequency (%), while continuous variables were reported as median (interquartile range). Chi-square and Student’s *t*-test were used for comparison of categorical and continuous variables, respectively. All statistical tests were two-sided and a level of 0.05 was used to indicate statistical significance. Data analysis was performed with the Statistical Software for the Social Sciences SPSS Advanced Statistics 22 (IBM Software Group, 200 W. Madison St., Chicago, IL; 60606 USA).

## Results

### Patients and clinical outcome

**Fig. 1:**
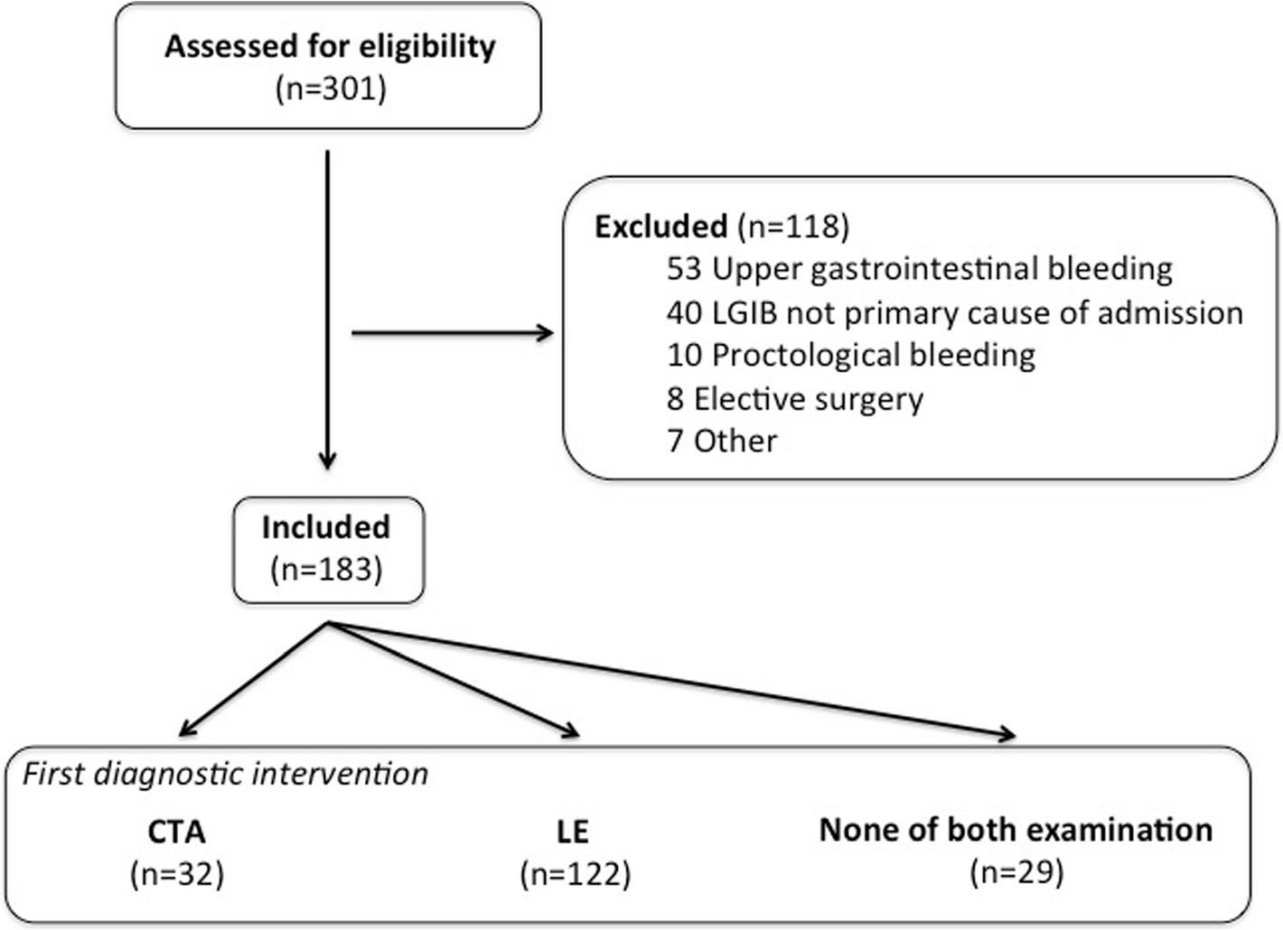
*Study flow chart.* Lower gastrointestinal bleeding (LGIB), Computed tomographic angiography (CTA), Lower endoscopy (LE)

Within the study period, 301 patients were hospitalized in the visceral surgery department with diagnostic of GIB. 118 patients were excluded according to the *a priori* defined rules. Final analysis included 183 patients, with 109 male and 74 female patients with a median age of 75 years (Fig. 1). One hundred and twenty-two patients (66.7%) had LE as first diagnostic intervention, 32 (17.5%) had CTA. The remaining 29 patients (15.8%) had neither of the two exams during their hospital stay. Patient characteristics are presented in Table 1. Median length of hospital stay (LOS) was 5 (IQR = 7) days. In the CTA group, LOS was longer compared to the LE group with 9 (IQR = 12.7) versus 5 (IQR = 6.6) days, respectively (*P* = 0.026). In-hospital mortality rate was 2.7% and concerned 5 patients. 3 patients died of postoperative complications (septic shock, intravascular disseminated coagulopathy, cardiac failure), one patient of multi-organ failure following hemorrhagic shock and one patient presented sudden cardiac arrest. Four out of these 5 deceased patients were examined with CTA first. Twenty-eight patients (15.3%) were referred after initial evaluation in a regional center. The CTA group had the higher proportion of referred patients (34.4%) compared with the LE group (9.8%). Patients’ hemodynamics are detailed in Table 1. MAP was significantly lower in the CTA group, compared with the LE group with 85 ± 18 versus 95 ± 16 mmHg, respectively (*P* = 0.006). Patients in the CTA group had similar Hb levels at admission compared with the LE group (102 ± 30 versus 112 ± 24 g/L, respectively; *P* = 0.09). SI was also similar in patients in the CTA group, with 0.72 ± 0.27 versus 0.63 ± 0.18 in those of LE group (*P* = 0.082). No significant difference was observed in the comparison of HR at admission in both groups (Table 1).

**Table 1:** Patient characteristics

	All patients (*n* = 183)	CTA (*n* = 32)	LE (*n* = 122)	*P*
Age, median (range)	75 (21–99)	68 (21–94)	77 (33–99)	***0.004***
Male, n (%)	109 (59.6)	19 (59.4)	71 (58.2)	*0.909*
Hb (g/L), Mean ± SD	110 ± 27	102 ± 30	112 ± 24	*0.090*
MAP (mmHg), Mean ± SD	93 ± 18	85 ± 18	95 ± 16	***0.006***
HR, Mean ± SD	83 ± 16	82 ± 15	83 ± 16	*0.881*
SI, Mean ± SD	0.66 ± 0.22	0.72 ± 0.27	0.63 ± 0.18	*0.082*
*Diagnostic, n (%)*				
Diverticular	48 (26.2)	9 (28.1)	38 (31.1)	*0.741*
Unknown location	41 (22.4)	5 (15.6)	21 (17.2)	*0.831*
Colo-rectal lesion	34 (18.6)	5 (15.6)	27 (22.1)	*0.419*
Post-interventional	23 (12.6)	2 (6.2)	17 (13.9)	*0.239*
Small bowel	20 (10.9)	11 (34.4)	4 (3.3)	***<0.001***
Colo-rectal neoplasia	17 (9.3)	0 (0)	15 (12.3)	***0.037***

Comparison of baseline characteristics of patients who had CTA and patients who had LE. Significant *P*-values (<0.05) are indicated in bold characters. SI is defined as HR/Systolic blood pressure

*CTA* Computed tomographic angiography*, LE* Lower endoscopy*, Hb Hemoglobin level, MAP* Mean arterial pressure*, HR* Heart rate*, SI* Shock index

### Radiological and endoscopic findings

**Fig. 2:**
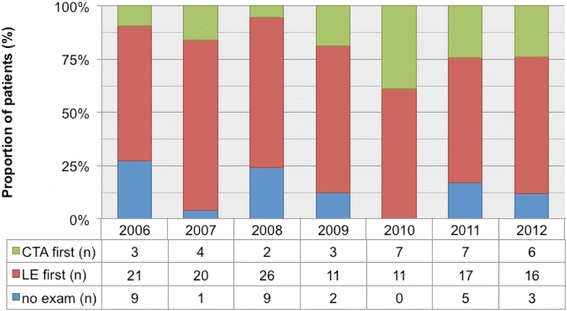
*Proportion of first diagnostic intervention per year.* Computed tomographic angiography (CTA), Lower endoscopy (LE)

In the first 3 years of the study period, LE was the predominating diagnostic tool, while CTA gained of importance in the second part of the study period only (Fig. 2).

Following hospital admission, CTA was performed significantly earlier than colonoscopy, after a median of 3 (IQR = 8.2) versus 22 (IQR = 36.9) hours (*P* < 0.001).

Active bleeding was found significantly more frequently with CTA compared to LE (31.3 *vs.* 14.8%, *P* = 0.031). A non-active bleeding source was identified in 21.8 *vs.* 31.1% by CTA and LE, respectively (*P* = 0.305). The rate of inconclusive exams was similar in both groups (46,9 *vs.* 54.1%, *P* = 0.396).

Patients presenting post-interventional bleeding were mostly evaluated by LE first, and rarely by CTA first (73.9 *vs.* 8.7%, respectively). Patients with small bowel bleeding underwent predominantly CTA first (55%) compared to LE first (20%), this difference was statistically significant (*P* <0.001).

### Bleeding control

**Fig. 3:**
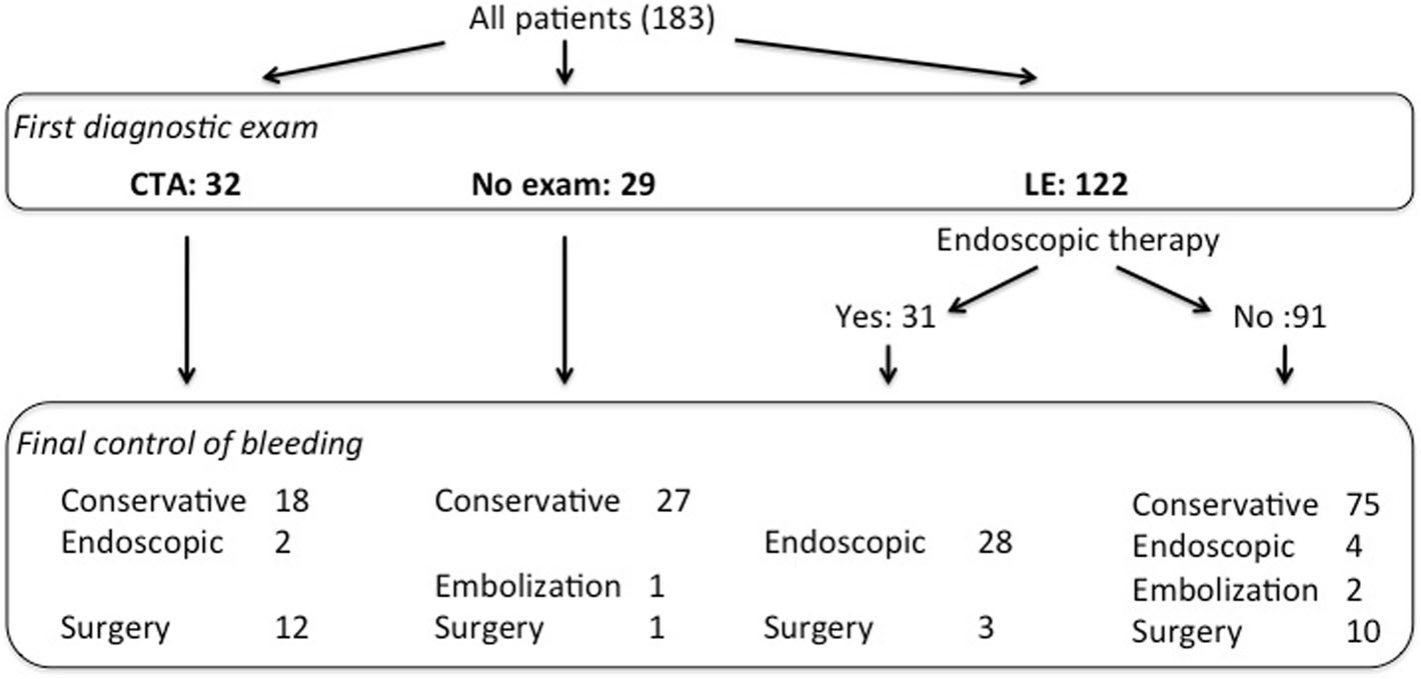
*Flow chart of the bleeding control according to the first exam used.* Values are presented as number of patients. Computed tomographic angiography (CTA), Lower endoscopy (LE)

**Fig. 4:**
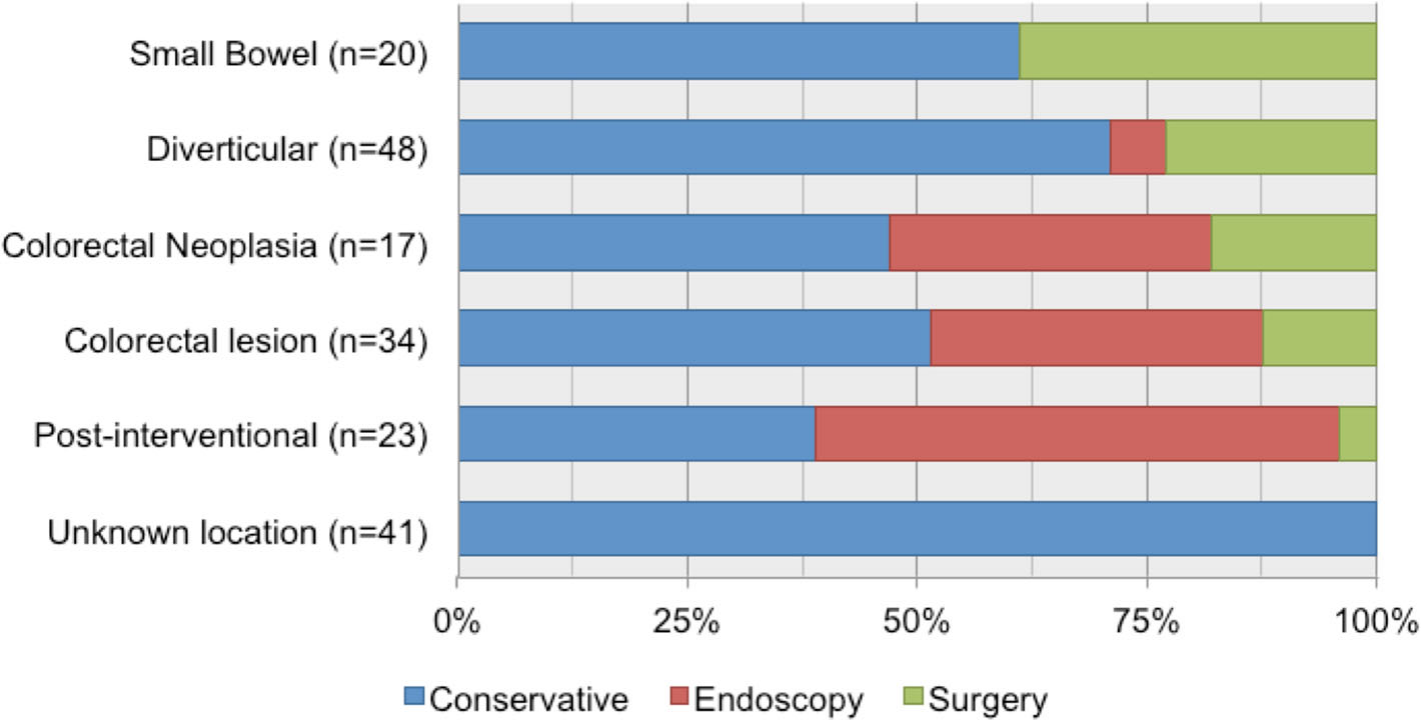
*Bleeding control according to the final diagnostic*

The control of bleeding was achieved by conservative measures in 120 (65.6%) patients, by endoscopic intervention in 34 (18.6%), by surgery in 26 (14.2%) and by embolization in 3 patients (1.6%), respectively. There were no differences in the rate of conservatively managed patients in the CTA group compared to the LE group (56.3 *vs.* 61.5%, *P* = 0.591). A summary of the final bleeding control according to the first exam used is shown in Fig. 3. Active bleeding on CTA was found in 10 patients and surgery was needed for the final control of the bleeding in 80% of cases, whereas the remaining 20% of patients were treated conservatively. After positive CTA for these 10 patients, 4 underwent surgery directly, 3 LE and 3 angiography. All 3 LE were inconclusive because of impaired vision due to blood clots, and only 1 out of 3 angiographies was positive allowing embolization in one single patient who eventually needed surgery for re-bleeding. In all these cases except for one, the bleeding localization was confirmed by the pathology report. Patients presenting post-interventional bleeding were primarily controlled by LE in 57%. Patients with small bowel bleeding needed more often surgery (35%) compared with other pathologies (Fig. 4). Of the 29 patients with no exam during hospital stay, 27 patients had conservative treatment and 2 underwent immediate invasive treatment due to hemodynamic instability. Embolization was performed for 1 patient and 1 patient underwent surgery directly. The remaining 27 patients were not further examined because of clinically minor bleeding, with spontaneous resolution.

## Discussion

The results of this study suggest that CTA may be a good choice in most patients with LGIB despite the fact that LE was the most used modality in our series and remains best choice for post-interventional bleeding. CTA was quickly available, reliable to indentify the bleeding source and helpful to guide further management.

In the group of patients assessed by CTA first, a significantly greater rate of active bleeding was observed compared to patients examined with LE first (31.3 *vs.* 14.8%, respectively, *P* = 0.031). While the shorter waiting time in the CTA group very likely increased the chance to identify actively bleeding lesions, the delay taken to perform LE increased the chance for spontaneous bleeding cessation. Delay in performing LE is explained by prior bowel preparation and limited gastroenterology consultant availability out of office hours. Nonetheless, about half of the exams were inconclusive in both groups, without significant difference. In a study including 115 patients with LGIB who underwent CTA, Chan & al. found that 77% of patients with negative studies did not need further intervention. In 68% of cases the exam did not show features of active bleeding, which is consistent with the present findings [7].

In the literature, the optimal time point of colonoscopy for LGIB remains controversial. In a retrospective analysis, Strate & al. revealed that shorter time to colonoscopy was an independent predictor of shorter LOS, particularly if colonoscopies were performed within 12–24 h [8]. A further trial confirmed that the source of bleeding was more frequently found with urgent colonoscopy (within 8 h) compared to elective colonoscopy (within 4 days) [9]. On the other hand, in a randomized trial comparing colonoscopies performed within 12 h of presentation to those executed within 36–60 h, the authors did not show differences in clinical outcomes. However non-diagnostic colonoscopies were more common in the elective group [10].

In this series, 65.6% of patients had spontaneous cessation of bleeding regardless of the first diagnostic exam chosen. This suggests that most patients presenting with LGIB can be managed conservatively. In the literature, self-limiting LGIB rates of up to 80% have been reported, especially in case of diverticular bleeding [4, 11, 12]. Surgery was required in 14% of patients in this serie which is similar to the published range of 2.6 to 18% [8, 9, 13, 14]. In-hospital mortality was 2.7% in the present study. Previously published mortality rates ranged from 2.4 to 8.8%, [3, 8, 13, 15].

The present results suggest that most patients with post-interventional bleeding were effectively treated with colonoscopy. In this sub-group of patients, examination with CTA was not necessary, and patients should directly undergo early colonoscopy to confirm diagnosis and deliver treatment at the same time. In the literature, several authors reported successful endoscopic management of post-polypectomy bleeding for most patients [16–18]. In the present series, 80% of patients with active bleeding on CTA required surgery for bleeding control. This is in line with Chan & al. reporting about 90% of patients with LGIB and positive CTA needing intervention for bleeding control. However, surgery was performed in 24% of cases whereas embolization was successful in 64% of patients [7]. In a study from Nagata & al., early colonoscopy following CTA resulted in a higher detection rate of colonic vascular lesions than colonoscopy alone [19]. These results contrast the present findings were all 3 LE performed after positive CTA were negative due to impaired visualization. Koh & al. suggested that angiography should be performed as soon as possible after positive CTA to allow embolization [20]. In their series, angiography was performed only after CTA with signs of active bleeding and about half of them were negative. In the present study, only 3 patients underwent angiography after positive CTA, 2 of them were negative and one patient underwent embolization first, followed by salvage surgery because of early re-bleeding.

**Fig. 5:**
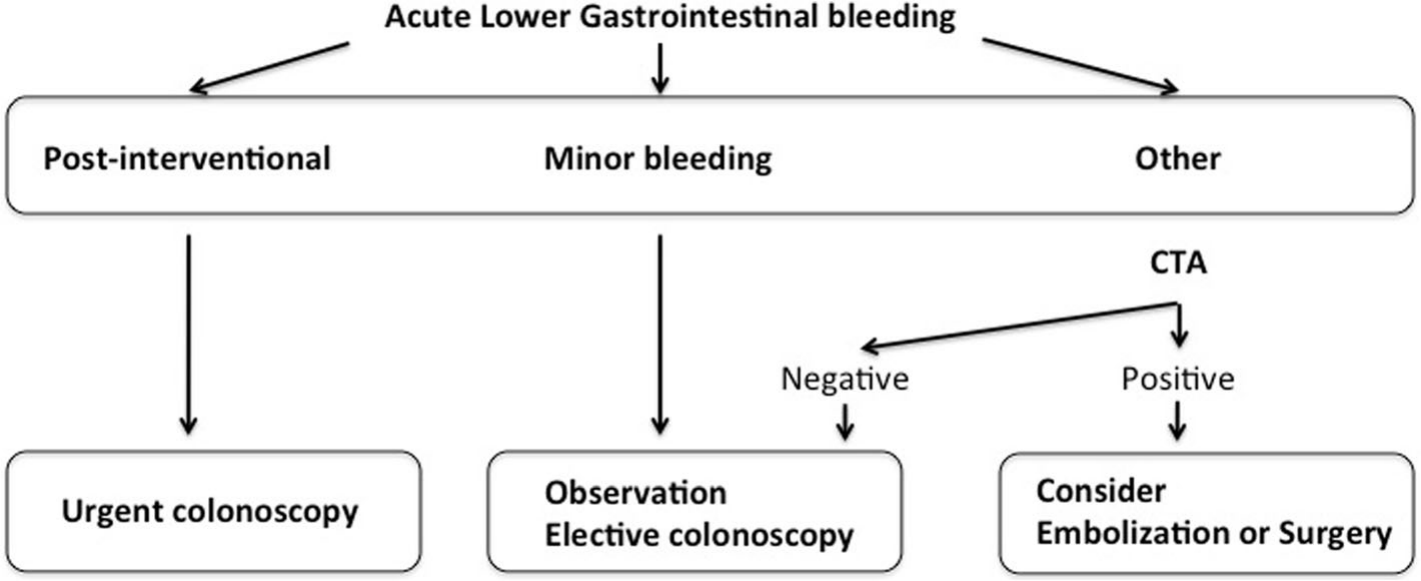
*Management of acute lower gastrointestinal bleeding, proposed algorithm.* Minor bleeding is defined as patients presenting without signs of hemodynamic comprise, need for transfusion or ongoing bleeding. Computed tomographic angiography (CTA)

Based on these findings, our own institutional algorithm for the management of LGIB was adapted (Fig. 5). For post-interventional bleeding, urgent LE is advocated. Patients presenting with minor bleeding can be observed and prepared for elective colonoscopy within 24 h. Patients presenting with more significant bleeding should undergo CTA as first-line procedure. It is hypothesized that this algorithm may help to decrease time to diagnosis and guide successful treatment. In published practice guidelines algorithms, radionuclide red blood cell scan followed by mesenteric angiography is indicated for patients unfit for colonoscopy or those with failed endoscopic therapy, but use of CTA is not included [1, 4]. Other authors have integrated CTA in their management algorithm. Copland & al. proposed, for clinically active bleeding, CTA as first-line procedure after exclusion of UGIB with nasogastric lavage. Colonoscopy is then performed if CTA localized the bleeding [21]. Another report proposed an algorithm including CTA for all patients with LGIB [22]. Chan & al. proposed a management pathway including CTA after negative endoscopic evaluation [7]. The fact that so much various algorithms are proposed suggests that further research is needed.

This study has several limitations besides its retrospective design and a limited number of patients. Within the study period, there was a change in care providers and hence in practice. Furthermore, the choice of the first diagnostic exam was decided by the treating physician. Significant lower MAP and trends to lower Hb level and higher SI indicate a selection bias as the severely affected patients were more likely to undergo CTA first. Longer LOS in the CTA group also probably reflects the greater severity of the bleeding. Patients’ co-morbidites, medication, medical or surgical history also probably led to a selection bias in the choice of the first exam. The retrospective nature of the study precluded us to ensure a valid comparison. We could not account for the delay in performing LE during the night, as the gastroenterology consultant’s availability was lower outside office hours.

## Conclusion

In conclusion, our analysis describes current practice of management for LGIB in a “real-world” setting in order to propose a new treatment algorithm that needs to be evaluated prospectively. The present study suggests that CTA is an efficient and readily available tool to manage patients with LGIB. CTA could be considered a suitable first diagnostic option for acute LGIB except for post-interventional bleeding which should entail immediate LE. However, most patients with LGIB can be treated conservatively.
